# Particle and gas phase sampling of PCDD/Fs and dl-PCBs by activated carbon fiber and GC/MS analysis

**DOI:** 10.1007/s11356-023-27052-8

**Published:** 2023-04-20

**Authors:** Marina Cerasa, Ettore Guerriero, Catia Balducci, Alessandro Bacaloni, Piero Ciccioli, Silvia Mosca

**Affiliations:** 1 Italian National Research Council, Institute of Atmospheric Pollution Research, Area Della Ricerca Di Roma 1, 00010 Montelibretti (RM), Italy; 2 Chemistry Department, Mathematics, Physics and Natural Sciences Faculty, Sapienza University Piazzale Aldo Moro, 5, 00185 Rome, Italy; 3 Italian National Research Council, Institute for Biological Systems, Area Della Ricerca Di Roma 1, 00010 Montelibretti (RM), Italy

**Keywords:** Air sampling, Gas/particle partitioning, PCDD/Fs, Dl-PCBs, Activated carbon fiber (ACF), Validation, GC/MS, Ambient air

## Abstract

Polychlorodibenzo-*p*-dioxins (PCDDs), polychlorodibenzofurans (PCDFs), and polychlorobiphenyls (PCBs) are semi-volatile compounds and can be partitioned in the atmosphere between the gas and particulate phase, due to their physicochemical properties. For this reason, the reference standard methods for air sampling include a quartz fiber filter (QFF) for the particulate and a polyurethane foam (PUF) cartridge for the vapor phase, and it is the classical and most popular sampling method in the air. Despite the presence of the two adsorbing media, this method cannot be used for the study of the gas-particulate distribution, but only for a total quantification. This study presents the results and the performance aim to validate an activated carbon fiber (ACF) filter for the sampling of PCDD/Fs and dioxin-like PCBs (dl-PCBs) using laboratory and field tests. The specificity, precision, and accuracy of the ACF in relation to the QFF + PUF were evaluated through the isotopic dilution technique, the recovery rates, and the standard deviations. Then the ACF performance was assessed on real samples, in a naturally contaminated area, through parallel sampling with the reference method (QFF + PUF). The QA/QC was defined according to the standard methods ISO 16000–13 and -14 and EPA TO4A and 9A. Data confirmed that ACF meets the requirements for the quantification of native POPs compounds in atmospheric and indoor samples. In addition, ACF provided accuracy and precision comparable to those offered by standard reference methods using QFF + PUF, but with significant savings in terms of time and costs.

**Supplementary Information:**

The online version contains supplementary material available at 10.1007/s11356-023-27052-8.

## Introduction

Persistent organic pollutants (POPs) are ubiquitous contaminants frequently found in sediments, soil, fish, wildlife, human adipose tissue, serum, and milk (Hart and Pankow 1994; Lee and Jones 1999; Rodan et al. 1999; Bergknut et al. 2011). With respect to other environmental compartments, the atmospheric burden of POPs is relatively small, but the air is considered the most important vehicle for their global redistribution, especially considering the low solubility in water (Piazza et al. 2013). POPs have common physicochemical features such as resistance to chemicals and to biodegradation, high lipophilicity, and therefore, a tendency to bioaccumulate in adipose tissue, moreover, exhibits toxic effects on humans and wildlife (Rodan et al. 1999; Bergknut et al. 2011). As semi-volatile organic compounds, POPs are in the atmospheric environments in equilibrium in both the gaseous and particulate phases for temperatures above 0 °C (Lei and Wania 2004). In particulate matter, they are linked to the solid matrix by physical and chemical bonds (Hippelein and McLachlan 2000; Larsson et al. 2013; Wang et al. 2021). In this study, two classes of POPs are considered: dioxin-like polychlorinated biphenyls (dl-PCBs) and polychlorinated dibenzo-*p*-dioxins/polychlorinated dibenzofurans (PCDD/Fs). Due to the multiple equilibria that these POPs can have, ISO/DIS (ISO 2007a, b) and US-EPA (EPA 1999a, b) methods for the determination of PCDD/Fs and dl-PCBs in the atmosphere and indoor air sampling require a quartz fiber filter (QFF) to collect particle-bound contaminants, followed by a cartridge filled with a solid sorbent, usually polyurethane foam (PUF) or styrene–divinylbenzene polymer (i.e., XAD-2 resin) to collect the vapor phase (Kaupp and Umlauf 1992; Król et al. 2011; Degrendele et al. 2020; Wu et al. 2020; López et al. 2021). Despite the double adsorbent media, this sampling system cannot be used for a reliable estimation of the gas-particle partition of PCDD/Fs and dl-PCBs because it is subject to a number of several sampling artifacts. In particular, there may be an over or underestimation of POPs in particulate matter or gaseous fraction: (i) the gas phase compounds adsorbed on the particulate matter could be stripped from the QFF to the PUF cartridge enriching the gaseous fraction; (ii) the particulate matter on the QFF can adsorb some of the gaseous compounds during sampling. Very common is the loss of part of the samples due to a sampled volume greater than the breakthrough volume. To prevent this problem, backup filters consisting of PUF (Degrendele et al. 2020), a combination of XAD-2/PUF (López et al. 2021), or XAD-2 (Wu et al. 2020) have been added to the line of the sampling train. Although some authors have used data obtained with multiple sampling trains to estimate the amount of PCDD/Fs and/or dl-PCBs in the gas and particulate phase in it, as already mentioned, the error committed in this procedure is high. These system sets can only be used for a total estimation of the compounds in both phases (Kaupp and Umlauf 1992; Hart and Pankow 1994; Lee and Jones 1999; Barbas et al. 2018; Wu et al. 2020).

A more correct evaluation of the gas-particle distribution of these analytes was carried out using a denuder upstream of the adsorption train for the gaseous phase only and a second adsorbent (or a mixture of adsorbents) downstream for collecting the particulate (Kaupp and Umlauf 1992; Forbes 2020).

In this work, an activated carbon fiber (ACF) is proposed as a suitable single adsorbent for the total collection (both vapor and particle phase) of PCDD/Fs and dl-PCBs in atmospheric and indoor samples, meeting the requirement of international standard methods ISO and EPA.

Activated carbon fibers or fabrics (ACFs) are considered an advanced group of porous materials with many advantages over granular or powder-activated carbons. ACFs have an extremely high specific surface area (SSA) characterized by a uniform micropores distribution that is directly exposed to the surface (Lordgooei et al. 2001). An ACF felt already validated as a passive sampler for PCDD/Fs and PCBs in the aqueous matrix (Cerasa et al. 2020) was used, characterized by a high specific surface area (SSA) and microporosity distribution. The felt has a sufficient thickness and mechanical strength to fully retain fine atmospheric particles at the sampling rates normally used for their collection with a high-volume sampler while maintaining almost zero impedance. To date, the ACF has already been used for the sampling of these classes of compounds in the air only as a backup filter, in the queue of the QFF + PUF train (Anezaki and Yamaguchi 2011; Anezaki and Kashiwagi 2021), but not for sampling.

In this work, the tests that led to the validation of ACF as the only adsorbent for the evaluation of total PCDD/Fs and dl-PCBs in the atmosphere are presented. First, laboratory tests were carried out, evaluating the sampling efficiency for the gas phase with an ad hoc sampling train. Subsequently, the efficiency of the total sampling and the matrix effect was evaluated through real sampling. The method proposed with the ACF was compared with the reference method QFF + PUF. The tests were performed using isotopically labelled standard solutions, through which the R% was evaluated to consider the precision, repeatability, and selectivity of the method. The validation of ACF as an adsorbent material takes the requirements defined by ISO 16000–13 e 14 and EPA TO-4A and TO-9A as QA/QC parameters. A unified adsorbent method could allow a considerable saving in time and solvent consumption, not to be underestimated a simplification in analysis (sampling, extraction, and clean-up procedures).

## Material and methods

### Standards and solvents

All ^13^C-labelled standards of PCDD/Fs (EN1948-ES, EN1948-SS, and EN1948-IS) and dl-PCBs (WP-LCS, P48-SS, and WP-ISS) were purchased from Wellington Laboratories, Canada, (Tables S2 and S3, Supplementary Information). For all tests, three solutions of standard ^13^C_12_ (10 pg/µl) containing PCDD/Fs and dl-PCBs congeners were used, combining the previous solutions depending on the tests performed. They are distinguished according to the order of addition in the sample: (i) standard sampling solution (SS solution) added to the adsorbent medium before starting the sampling, (ii) extraction standard solution (ES solution) added to the adsorbents after sampling and before extraction, (iii) internal standard solution (IS solution) added before injection and used for quantification of native compounds and recovery rates (%R) of SS and ES solutions. The composition of the SS, ES, and IS solutions is specified later in the sections corresponding to each test. The GC/MS calibration was performed by the isotopic dilution method, using commercially available calibration curves: EN1948-CVS for PCDD/Fs and P48-W-CVS for dl-PCBs (Wellington Laboratories, Canada). Acetone, toluene, dichloromethane, and hexane used in chemical analysis were purchased from Romil.

### Activated carbon fiber (ACF)

The physico-chemical characterization of ACF (Chemical Research 2000 Srl, Italy) used in this work was already described in a previous study (Cerasa et al. 2020). Briefly, the Brunauner-Emmet-Teller (BET) method and the Langmuir equation were used to define an SSA of about 2500 m^2^/g with a pores diameter of ~ 1.2 nm (microporosity). The Boehm titration analysis yielded a strong acidic and basic component due to carboxyl and pyrone groups, respectively. All organics present on the ACF either due to material production or prolonged exposure to polluted atmospheres were removed by Soxhlet extraction with toluene for 24 h. The ACF was vacuum-dried at 40 °C prior to the use. It was cut into discs with a diameter of 58 and 102 mm, depending on the experiment.

### Sampling and collection trains used

**Fig. 1:**
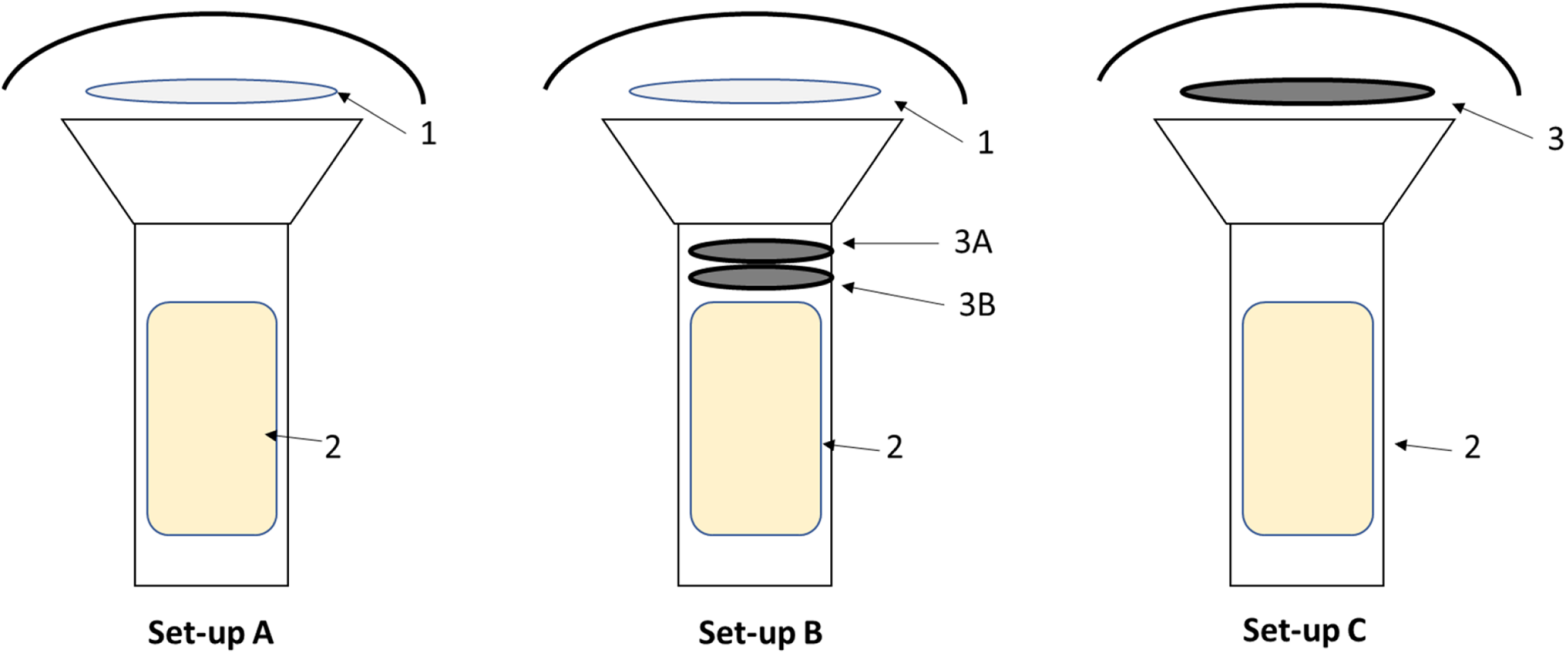
Sampling trains for the collection of PCDD/Fs and dl-PCBs used in this work. Set-up A: sampling train compliant with ISO and US-EPA standard methods for the collection of PCDD/Fs and dl-PCBs in the air (reference sampling method). 1, QFF (102 mm); 2, PUF; Set-up B: sampling train for ACF breakthrough test of PCDD/Fs and dl-PCBs. 1, QFF (102 mm); 3A, ACF (58 mm); 3B, ACF (58 mm); 2, PUF; Set-up C: sampling train to validate the use of ACF as a single sorbent for the determination of PCDD/Fs and dl-PCBs in indoor and ambient air samples; 3, ACF (102 mm); 2, PUF

High-volume samplers from TCR Tecora (Cogliate, Italy) with a PM10 cutting sampling head were used in all experiments. The sampling head includes a 102-mm grid holder to house a filter and a cylindrical glass cartridge holder (58 mm × 125 mm long) to house a polyurethane foam (PUF, density 0.022 g/cm^3^) plug. For all tests, a flow rate of 200 L/min was adopted and maintained constant based on temperature and atmospheric pressure through an electronic system. Figure 1 shows a scheme of the sampling train set-up, as described in detail in the following sections. Set-up A is the reference method, consisting of a QFF and a PUF. In Set-up B, two 58-mm ACF filters (3A and 3B) were placed in the glass cartridge, above the PUF. In Set-up C, the 102-mm QFF was directly replaced with a 102-mm ACF. Before sampling, the QFF was baked in an oven at 400 °C for 5 h, and the PUF was washed by Soxhlet extraction for 24 h with acetone: ethyl acetate (50:50 v/v). ACF was precleaned by 24 h-Soxhlet extraction with toluene and vacuum-dried at 40 °C prior the use.

#### Reference method

The reference method (Fig. 1, Set-up A) meets the requirements of both ISO (indoor air) and EPA (ambient air) standard methods for PCDD/Fs and dl-PCBs samplings (Table S1, Supplementary material). The sampling head includes a 102-mm QFF for the collection of particles and a PUF to collect the gaseous fraction. The SS solution (EN-1948SS and P48-SS, 100 µl; Tables S2 and S3, Supplementary material) was added to the QFF. After sampling, the QFF and the PUF were taken to the laboratory and extracted together in a cellulose thimble with ~ 3 g of Na_2_SO_4_, after adding the ES solution containing EN1948ES and WP-LCS (100 µl) (Tables S2 and S3, Supplementary material). The extraction was performed in 250-mL Soxhlet for 36 h with toluene. The extract was first concentrated with a rotary evaporator (40 ± 2 °C and 49 mbar) up to 10 ml and then with a gentle flow of N_2_ in a water bath (40 ± 2 °C) up to 1 ml. The clean-up involved a multilayer silica column (extract eluted with hexane) and an alumina microcolumn, to separate the PCDD/Fs from the dl-PCBs, as described in Mosca et al. (2010). The two fractions of the eluates were concentrated, and the corresponding IS solutions were added (EN 1948IS for PCDD/Fs and WP-ISS for dl-PCBs; Tables S2 and S3, Supplementary material). Instrumental analysis was performed using a triple quadrupole gas chromatograph/mass spectrometer (Trace 1310 GC/TSQ 8000 Evo, Thermo), and chromatographic separations were achieved using a DB-XLB column (60 m, 0.25 mm, 0,25 mm I.D., Agilent J&W) (Benedetti et al. 2017).

#### Evaluation of ACF breakthrough

A preliminary survey was conducted to investigate whether and to which extent PCDD/Fs and dl-PCBs can be retained in the vapor phase on the ACF sorbent at different sampled volumes, using ^13^C-labelled compounds as tracers, and the ISO breakthrough limits as a reference (ISO 2007a, b). The sampling train used for these tests consisted of a 102 mm QFF, two 58 mm ACFs (3A and 3B), and a PUF (Fig. 1, Set-up B). The QFF was spiked with a known amount of a mixture containing labelled congeners of both PCDD/Fs and dl-PCBs, used as a SS solution (1000–2000 pg of EN1948ES and 1000 pg of WP-LCS; Tables S2 and S3, Supplementary material), in order to simulate a real atmosphere sampling (ISO 2007b; Cerasa et al. 2020). The samplings were performed at different time extensions 24 h (288 m^3^), 3 days (864 m^3^), and 1 week (2016 m^3^), between March and May 2016 (detailed information in Table S4, Supplementary Material) in triplicate at the “A. Liberti” monitoring station of Montelibretti (Rome, Italy, located in the National Research Council of Italy (CNR)) classified as semiurban area, where the concentrations of native PCDD/Fs and dl-PCBs are usually below the limits of detection (LOD). After sampling, the QFF, the ACFs (3A and 3B), and PUF adsorbents were separately extracted with toluene in a Soxhlet apparatus for 36 h, once spiked each of them with the ES solution, containing1000 pg of P48-SS and 1000–2000 pg of EN-1948SS (Tables S2 and S3, Supplementary material).

The efficiency of the extraction of these classes of POPs from the ACF has already been investigated in previous studies (Cerasa et al. 2021). Separated fractions of PCDD/Fs and dl-PCBs were obtained by using the clean-up procedure described in the previous subsection. They were all fortified with IS solutions (1000 pg of WP-ISS and 1000 pg of EN 1948 IS, for dl-PCBs and PCDD/Fs, respectively. Tables S2 and S3, Supplementary material) before the GC–MS determinations.

#### ACF as a single sorbent

The suitability of ACF as a single absorbent for the determination of the total content (vapor and particulate phase) of PCDD/Fs and dl-PCBs in the air was assessed by seven parallel samplings collected between May and June 2016, sampling lasting from 24 to 168 h for total volumes between 480 m^3^ and 824 m^3^, in a very large indoor public area (> 50,000 m^3^) where a serious fire occurred causing emissions of black smoke particles, presumably enriched with PCDD/Fs and dl-PCBs, due to the presence of electric material (Colapicchioni et al. 2020).

The samples were collected in parallel on 102 mm QFF + PUF (Fig. 1 Set-up A), used as a reference according to the ISO/DIS standard methods (ISO 2007a, b) and US- EPA (EPA 1999c, b), and on 102-mm ACF+PUF (Fig. 1 Set-up C). In this sample train (ACF+PUF), the PUF acts as a backup filter for ACF, to verify the absence of a breakthrough in a contaminated atmosphere (matrix effect).

For this reason, ACF and PUF were extracted separately. Before sampling, the SS solution was added to the 102-mm ACF, then ACF and PUF were spiked with the ES Solution and extracted separately. The samples were then purified and the IS solution was added for GC–MS analysis. Standards and quantities added, purification method, and GC–MS analysis are the same as reported in the reference method subsection. Data concentrations of each congener of PCDD/Fs and dl-PCBs, expressed in fg TEQ/Nm^3^, were compared.

### Quality assurance/quality control

The validation of the proposed method based on ACF was carried out using the parameters defined by the standardized methods ISO 16000 13 and 14 and EPA TO 4A and 9A as QA/QC (Table 1).

**Table 1:** QA/QC acceptance criteria and requirements of ISO and EPA reference methods. *% R*_*SS*_, recovery rates of SS; *% R*_*ES*_, recovery rate of ES; ^1^ if the %R_SS_ is < 50 or > 150, the sampling is invalid

	*% R*_*SS*_	*% R*_*ES*_
*EPA TO 9A (PCDD/Fs)*	50–120	50–120 (TCDD/F, PeCDD/F, HxCDD/F) 40–120 (HpCDD/F, OCDD/F)
*EPA TO 4A (dl-PCBs)*	-	60–120 (PCB)
*ISO/DIS 16000–13*	(50) 75–125 (150)^1^	
*ISO/DIS 16000–14*	n.a	50–130 (TCDD/F, PeCDD/F, HxCDD/F) 40–130 (HpCDD/F, OCDD/F) 40–120 (PCB)

Since the recovery rate ranges imposed by EPA methods are stricter, they were taken as the QC acceptance criteria. The accuracy achieved for duplicates must be ± 30% (EPA 1999b). Furthermore, the breakthrough of the original sampling train shall be less than 10% for every single congener (ISO 2007a, 2007b).

All tests involved the use of isotopically labelled standards during all steps (the SS solution in the sampling step, the ES solution in the extraction step, and the IS solution before injection). The recoveries evaluated for all the analytical phases, allow us to interpret the losses of the compounds during each step and ensure the selectivity of the method.

The accuracy was estimated as the mean recovery rate of each labelled compound of the standard solutions (%R_SS_) spiked on the samples and the relative standard deviation (RSD%). The sampling efficiency is the accuracy during the sampling step evaluated through the recovery rate of the SS solution (%R_SS_) added before the sampling (Eq. 1).

1 $$\mathrm{\%}{R}_{SS}=\frac{RRF}{100}* \frac{{A}_{SS}}{{A}_{ES}}* \frac{{Q}_{ES}}{{Q}_{SS}}$$

The relative response factor (RRF) is the response of the mass spectrometer to a known amount of an analyte relative to a known amount of a ^13^C-labelled internal standard calculated through the calibration kit. A_SS_ and A_ES_ are the sums of the integrated ion abundances of the quantitation ions for ^13^C-labelled SS and ES solution compounds; Q_SS_ and Q_ES_ are the quantities of the ^13^C-labelled SS and ES solutions injected.

Furthermore, the accuracy of the ACF method was evaluated in relation to the reference method (QFF + PUF) by both considering the %Rs of all 7 parallels and comparing the quantitative analyses of each pair of congeners for a single sample. The standard deviation of the %R of the triplicates for the laboratory tests and the seven samples served as a measure of the method’s precision. Linearity is evaluated through tests to verify the ability of the ACF to adsorb the gas phase: sampling was carried out with progressively increased volumes of air while still using the same amount of spiked standards. The 7 real samplings performed in a heavily polluted area affected by a fire are accounted for the matrix effect, which is assessed using the average %R and RSD%.

A standard mixture of isotopically labelled PCDD/Fs and dl-PCBs was injected repeatedly throughout the batch to test the stability of the analytical instrument, and solvent blank injections (nonane) for GC analysis were used to track potential carry-over and memory effects. These procedures were done to investigate the instrument’s precision. All tests included a laboratory and a field blank for real samples. Generally, LODs and LOQs were calculated to check the sensitivity of the developed method for target compounds. LODs and LOQs were defined as 3 and 10 times the signal-to-noise ratio (S/N) under the lowest spiked concentration of the calibration curve, respectively.

## Results and discussion

Peters et al. (2000) have shown that release of POPS occurs from particles during sampling, as a function of the partial vapor pressure of the specific POP and the volume sampled. According to Peters, these vapors are transferred to the solid adsorbent placed after the particle filter, where they should be retained. Since the retention of PCDD/Fs and dl-PCBs on the QFF + PUF sampling train depends on their concentration in air, the environmental conditions in which sampling is performed, and the total volume sampled, adequate quality control and quality assurance criteria (QA/QC) were defined to attest that an accurate determination in the air is achieved. It can happen, as one of the most common reasons, when the sampled volume exceeds the breakthrough volume on the PUF adsorbent, and hence, part of the sample is lost during sampling. The following subsection argues the cited QA/QC criteria.

### Retention of PCDD/Fs and dl-PCBs in the vapor phase on the ACF

First of all, the retention of PCDD/Fs and dl-PCBs congeners on the ACF was investigated. The sampling train used consisted of the following: 102-mm QFF; two-58 mm AFCs (3A and 3B) and a PUF (Fig. 1, Set-up B), collecting samples at three different volumes, up to 2016 m^3^ and by spiking the QFF with appropriate amounts of a SS solution, as described in the corresponding section. The spiking approach is simple and provides results that can be safely extrapolated to a real atmospheric sampling, because the retention volume measured is equal to or smaller than that measured under normal atmospheric sampling conditions. Since the most volatile fraction of the SS solution is rapidly stripped from the QFF, a nearly instantaneous transfer to the ACF 3A adsorbent occurs as soon as the aspirating pump is activated. This effect does not normally occur under atmospheric sampling conditions because the stripping of semi-volatile POPs from particles retained on the QFF is much slower, and larger volumes are required to let POPs vapor them to pass through the ACF 3A adsorbent. Increasing volumes were sampled to check the linearity and to see if the breakthrough volume of PCCD/Fs and dl-PCBs congeners was ever reached on the ACF 3A adsorbent in 168-h samples.

**Fig. 2:**
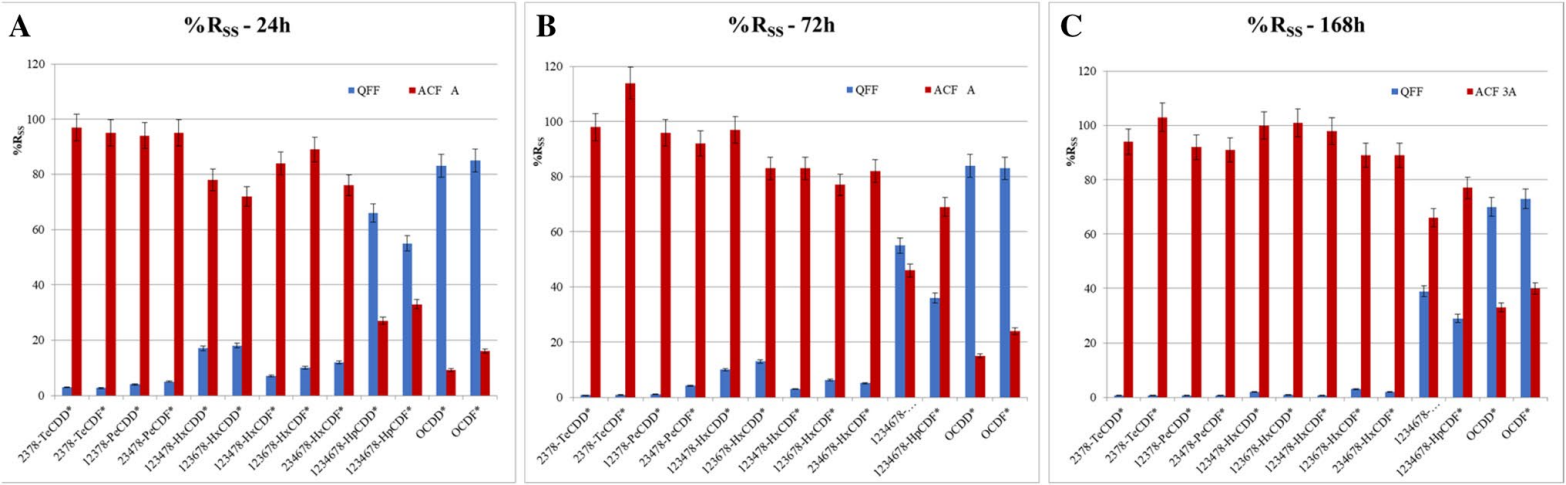
Comparison of average %R_SS_ on 102 mm QFF and 58 mm ACF 3A for PCDD/Fs (Set-up B), at different sampling times (and corresponding volumes). **A** 24 h (288 m^3^); **B** 72 h (876 m^3^); **C** 168 h (2016 m^3^). The “*” means ^13^C-labelled

**Fig. 3:**
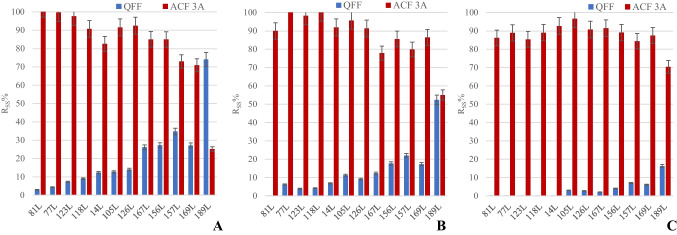
Comparison of average %R_SS_ on 102 mm QFF and 58 mm ACF 3A for dl-PCBs (Set-up B), at different sampling times (and corresponding volumes). **A** 24 h (288 m^3^); **B** 72 h (876 m^3^); **C** 168 h (2016 m.^3^). The individual PCB congeners are reported according to the IUPAC nomenclature; the “L” means “labelled”

Since the atmospheric concentration of the contaminants of interest for this paper (native compounds) in “A. Liberti” monitoring station is below the limit of quantification, and labelled compounds act as the natives, a simulated polluted air with a known amount of ^13^C labelled SS solution (ISO 2007b; Cerasa et al. 2020) spiked on the QFF was used. All the sorbents were separately extracted, and recovery rates of SS solution (%R_SS_) were evaluated for each sampling test. The breakthrough volume of the ACF 3A could be considered analytically insignificant since the %R_SS_ in ACF 3B and in PUF were lower than 10% of the initial amount spiked on QFF. Figures 2 and 3 report the average recovery rates of SS solution (%Rss) of triplicate sampling for PCDD/Fs and dl-PCBs on QFF and ACF 3A adsorbent for the sampling volumes of 288, 876, and 2016 m^3^. The %R_SS_ of QFF, ACF A, and ACF B in every test are presented in Supplementary Material, Tables S5–S7. The fraction collected on PUF was always < LOD.

The analysis of data in Figs. 2 and 3 shows that the partitioning of PCDD/Fs and dl-PCBs congeners between the QFF and the ACF 3A adsorbent is fully coherent with the values of their partial vapor pressure (Peters et al. 2000) that, in the homologous series investigated, is inversely related to the number of chlorine atoms in the molecule and molecular weight. Concerning PCDD/Fs (Fig. 2), the fraction of tetrachloro-substituted PCDD/Fs retained on the QFF was < LOD, whereas that of octachloro-substituted PCDD/F was still ca. 70%, at the maximum sampled volume (2016 m^3^). Differences in the partial vapor pressure also explain why PCDFs with an increasing content of chlorine atoms in the molecule were less retained on the QFF than the corresponding PCDDs congeners having the same degree of chlorination. Similar considerations apply for dl-PCBs reported in Fig. 3, where the most volatile congeners, such as the tetra- and penta-chlorinated ones, were completely lost from the QFF after 24-h of sampling (Fig. 3 A), whereas ca. 16% of the hepta- congeners was still present in it after 168 h of sampling (Fig. 3 C). As expected, an increase in the sampled volume produced an increasing release of PCDD/Fs and dl-PCBs from the QFF, that were transferred as vapors to the ACF 3A adsorbent. Tables S5–S7 in the supplementary show the concentration of PCDD/Fs and dl-PCBs identified separately on each sorbent.

The congeners of PCDD/Fs and dl-PCBs collected in the backup filter ACF 3B and in the PUF were almost all < 5% of the initial amount spiked on the QFF. This confirms the absence of breakthrough for up to 2000 m^3^ sampled and that one 58-mm ACF filter can retain the analytes investigated.

### Collection of total PCDD/Fs and dl-PCBs in the air on a single ACF sorbent

Since no breakthrough volume was achieved on the ACF by any of the tested PCDD/Fs and dl-PCBs congeners, the adsorbent has been shown to efficiently collect the gas phase. Then, the adsorption/retention efficiency of ACF of the particle-bound POPs, introducing the matrix effect was evaluated. As described in the “ACF as a single sorbent” subsection, air samples were collected on a sampling train consisting of a 102-mm ACF/PUF (Fig. 1, Set-up C) in parallel to the reference 102-mm QFF + PUF system (Fig. 1, Set-up A), in an environment naturally contaminated by PCDD/Fs and dl-PCBs. The PUF was considered only as a backup filter. The first step was to define the validity of the parallel samplings (ACF vs QFF + PUF) according to previously reported QA/QC. For this purpose, the %R_ES_ were considered and evaluated: if they fall within the established ranges, it means that none of the laboratory steps (extraction and clean up) affects the sample. Once the losses due to the processes that the sample undergoes in the laboratory had been evaluated, the %R_SS_ were considered. Tables 2 and 3 report the average (*n* = 7) and the range of %R for ES and SS, obtained for the labelled PCDD/Fs and dl-PCBs, respectively.

**Table 2:** Mean values of %R_ES_ and %R_SS_ of PCDD/Fs (*n* = 7) for samples collected with the reference method (QFF + PUF, Set-up A) and the ACF (Set-up C). The PUF in the Set-up C was added as a backup filter

	Set-up A		Set-up C			
	QFF + PUF		ACF		Backup PUF	
	Mean	Min–max	Mean	Min–max	Mean	Min–max
%R_ES_						
^13^C-2378-TeCDD	69	52–78	70	54–76	76	51–81
^13^C-12378-PeCDD	79	51–88	83	50–106	97	70–117
^13^C-123478-HxCDD	60	60–68	70	62–72	60	59–72
^13^C-123678-HxCDD	64	52–70	73	59–93	91	59–109
^13^C-123789-HxCDD	49	50–100	76	58–87	67	63–87
^13^C-1234678-HpCDD	92	50–111	96	65–113	73	65–81
^13^C-OCDD	78	53–93	80	59–88	89	59–92
^13^C-2378-TeCDF	87	53–98	108	53–114	82	79–91
^13^C-12378-PeCDF	90	57–119	88	67–114	76	69–84
^13^C-23478-PeCDF	91	62–118	83	64–113	74	70–79
^13^C-123478-HxCDF	84	60–96	95	69–108	101	73–102
^13^C-123678-HxCDF	99	64–88	75	69–79	77	72–80
^13^C-234678-HxCDF	105	62–84	82	61–121	95	65–99
%R_SS_						
^13^C-12378-PeCDF	92	83–106	102	69–114	< 1	-
^13^C-123789-HxCDF	74	61–87	76	76–87	< 1	-
^13^C-1234789-HpCDF	90	84–98	87	81–105	< 1	-

**Table 3:** Mean values of %R_ES_ and %R_SS_ of dl-PCBs (*n* = 7) for samples collected with the reference method (QFF + PUF, Set-up A) and the ACF (Set-up C). The PUF in the Set-up C was added as a backup filter

	Set-up A		Set-up C			
	QFF + PUF		ACF		Backup PUF	
	Mean	Min–max	Mean	Min–max	Mean	Min–max
%R_ES_						
81L	90	88–112	92	91–94	76	70–85
77L	90	84–114	100	75–104	72	64–82
123L	85	71–105	76	77–96	80	77–82
118L	76	67–95	70	72–91	79	77–82
114L	72	64–89	67	66–102	93	63–113
105L	69	67–82	73	67–76	79	72–83
126L	65	64–73	73	66–78	73	70–76
167L	108	79–116	88	68–121	85	71–88
156L	109	82–118	80	72–93	78	69–79
157L	103	91–116	82	78–103	90	68–96
169L	79	86–105	80	76–84	58	63–95
189L	92	87–94	88	79–94	83	71–86
%R_SS_						
60L	134	69–137	103	79–121	16	< 1–23
127L	89	81–92	83	79–91	< 1	< 1–3
159L	53	51–54	54	51–56	< 1	< 1

An analysis of data (Tables 2 and 3) shows that results obtained on the single 102-mm ACF filter were comparable to those obtained by collecting PCDD/Fs and dl-PCBs on the combined QFF + PUF reference sampling train. Recovery rates on the backup PUF showed that only limited amounts of PCDD/Fs and dl-PCBs congeners were released from the ACF filter, with a highest value of 16% reached by the most volatile 2,3,4,4′-tetra-CB (60L, sampling standard). Despite the lower %R_SS_ of 159L (2,3,3′,4,5,5′-hexa-CB), the values fall within the second range of sampling efficiency between 50 and 150% still considered valid (ISO 2007a). %R_SS_ values of 159L were systematically lower than those measured in the experiments performed in the “A. Liberti” monitoring station. Since this effect was independent of the sampling train used, it was most likely caused by the different nature and concentrations of POPs collected in the particle and gas phases in the two experiments. The HxCDD/F congeners likewise had a lower extraction efficiency when compared to earlier studies, showing a similar effect. Since the determination of native compounds was possible in all samples, possible matrix effects arising from changes in the sample composition and POP concentrations were investigated. Comparing the concentrations of native PCDD/PCDF and PCB congeners determined with ACF and the QFF + PUF combination when different volumes were passed to the sampling trains would have allowed for the detection of matrix effects, if they had occurred.

The minimum and maximum recoveries of all the samples for each class of PCDD/Fs and dl-PCBs fulfil the extraction and sampling efficiency requirement of ISO 16000–13 and 14 and EPA TO-4A and TO-9A reference methods. These results demonstrate that both adsorption sampling trains, QFF + PUF and ACF, are accurate in sampling micropollutants from both outdoor and indoor air. The recoveries of backup PUF corroborate the validity of the results demonstrating the absence of a breakthrough volume since the %R_SS_ are less than 10% of the total initial amount added on ACF (Tables 4 and 5). The %R_ES_ are all within the range, validating the results of %R_SS_. The determination of native compounds was made possible because all seven parallels met the QA/QC standards (%R_ES_ and %R_SS_) and could be considered valid. Tables 4 and 5 report only the concentrations (fg TEQ/m^3^) of native PCDD/PCDFs and dl-PCBs congeners, respectively, at two different sampling volumes (480 and 830 m^3^) on the two set-ups: reference method (QFF + PUF) and the proposed single ACF filter, with a backup PUF.

**Table 4:** Comparison of PCDD/Fs concentrations in fg TEQ/Nm^3^ between the reference method (QFF + PUF) and the proposed one (ACF and PUF as a backup adsorbent). Sample A = 480 m^3^ ~ 48 h; Sample B = 830 m^3^ ~ 72 h

PCDD/Fs	**Sample A (480 m**^**3**^**)**			**Sample B (830m**^**3**^**)**		
fg TEQ/Nm^3^	QFF + PUF	ACF	Backup PUF	QFF + PUF	ACF	Backup PUF
2378-TeCDD	24.0	28.7	0.2	49.3	47.0	0.1
12,378-PeCDD	20.1	24.5	0.9	13.3	9.5	0.1
123,478-HxCDD	0.05	1.0	0.05	0.03	0.01	0.02
123,678-HxCDD	0.3	0.6	0.05	0.1	0.7	0.02
123,789-HxCDD	0.06	0.1	0.05	0.08	0.1	0.01
1,234,678-HpCDD	0.04	0.02	0.02	0.07	0.04	0.01
OCDD	0.02	0.01	0.001	0.004	0.03	0.001
2378-TeCDF	1126	1201	17.3	1284	1324	16.2
12,378-PeCDF	96.2	72.6	0.8	106.3	72.5	0.01
23,478-PeCDF	287.4	311.5	0.8	554.2	485.2	9.0
123,478-HxCDF	37.0	23.3	0.6	50.5	66.3	0.8
123,678-HxCDF	35.2	26.3	0.03	29.6	37.1	0.3
234,678-HxCDF	8.3	13.6	0.3	5.0	5.9	1.0
123,789-HxCDF	2.0	1.7	0.04	1.8	1.7	0.02
1,234,678-HpCDF	2.9	2.1	0.3	3.7	1.7	0.1
1,234,789-HpCDF	0.2	0.3	0.003	0.8	0.3	0.001
OCDF	0.001	0.003	0.001	0.02	0.04	0.004
**Total PCDD/F**	**1639.8**	**1707.3**	**21.4**	**2098.8**	**2052.5**	**27.6**

**Table 5:** Comparison of dl-PCBs concentrations in fg TEQ/Nm^3^ between the reference method (QFF + PUF) and the proposed one (ACF and PUF as a backup adsorbent). Sample A = 480 m^3^ ~ 48 h; Sample B = 830 m^3^ ~ 72 h

dl-PCBs	**Sample A (480 m**^**3**^**)**			**Sample B (830m**^**3**^**)**		
fg TEQ/Nm^3^	QFF + PUF	ACF	Backup PUF	QFF + PUF	ACF	Backup PUF
PCB 81	1.2	1.7	< LOD	1.4	1.7	< LOD
PCB 77	6.1	6.4	< LOD	7.0	7.0	< LOD
PCB 123	4.2	4.4	< LOD	5.4	5.3	< LOD
PCB 118	47.2	45.0	< LOD	62.4	59.0	< LOD
PCB 114	1.3	1.2	< LOD	1.3	1.4	< LOD
PCB 105	13.8	13.1	0.1	18.4	18.1	0.1
PCB 126	8.2	9.7	1.3	1.3	3.2	1.2
PCB 167	0.9	1.1	< LOD	1.1	1.0	< LOD
PCB 156	2.1	2.1	< LOD	2.3	2.0	< LOD
PCB 157	< LOD	< LOD	< LOD	< LOD	< LOD	< LOD
PCB 169	76.2	82.1	1.2	12.3	15.6	0.8
PCB 189	< LOD	< LOD	< LOD	< LOD	< LOD	< LOD
**Total dl-PCBs**	**161.2**	**166.8**	**2.6**	**112.9**	**114.3**	**2.1**

Quantitative analysis of native compounds for the seven parallel samplings confirms what was observed by the %Rss value. The matrix effect and the high concentrations of the contaminants in the air appear to be the key influencing factors in the sampling. For this reason, the higher and the lower air volume samplings were compared in Tables 4 and 5.

The results (Tables 4 and 5) show that a close correlation existed between the concentrations of native PCDD/Fs and dl-PCBs congeners measured with the two sampling methods. This implied that matrix effects caused by the ACF were minimal and that, regardless of the volume sampled, a strong correlation between the two data sets was feasible. Pearson’s correlation coefficient, evaluated for ∑ PCDD/Fs and ∑ dl-PCBs in fg TEQ/Nm^3^ from the seven parallel samplings, resulted in 0.927 and 0.892, for ∑ PCDD/Fs and ∑ dl-PCBs, respectively, confirming a strong correlation between the two sampling systems.

**Fig. 4:**
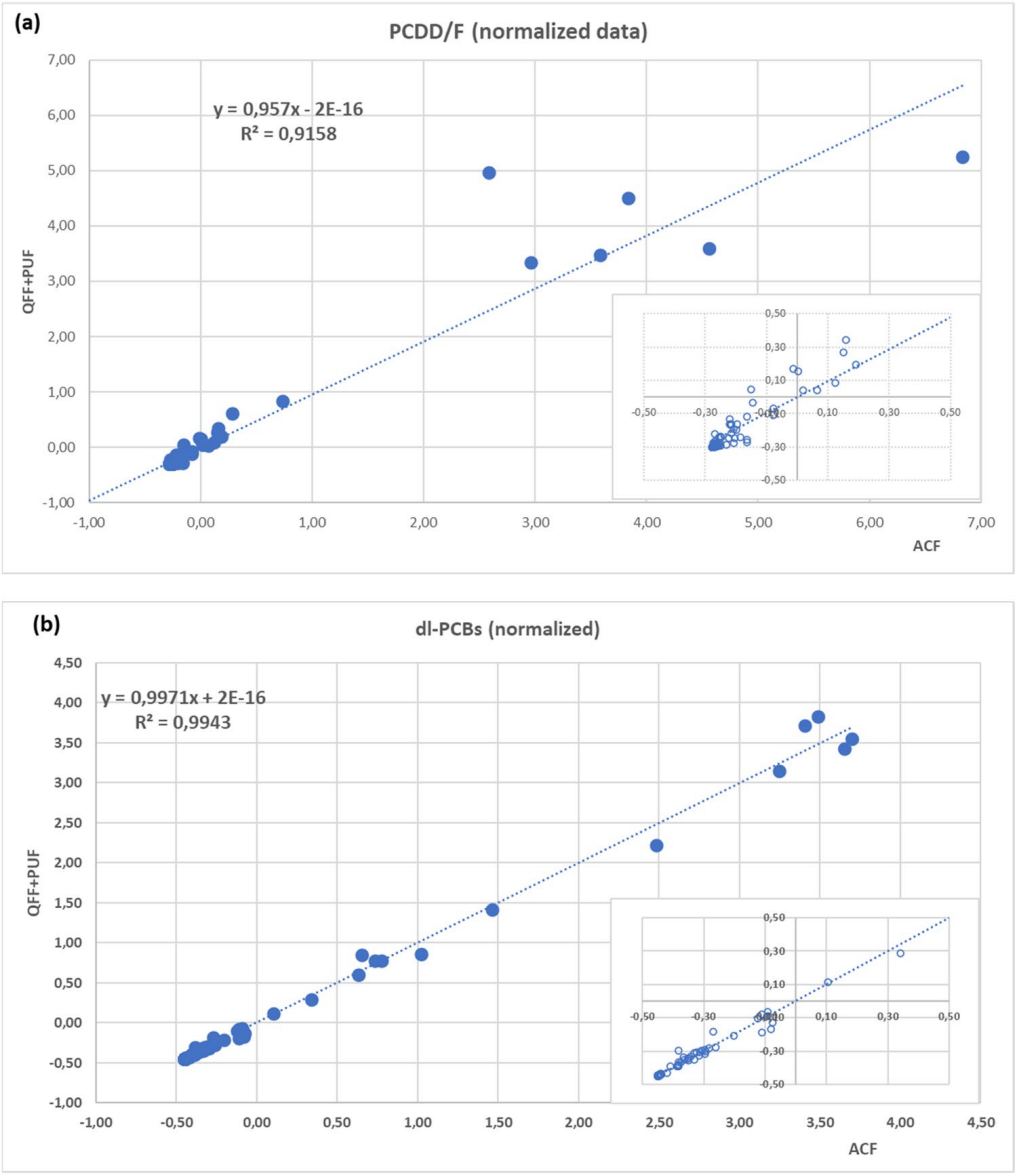
Normalized data concentrations of each congener from seven parallel samplings. Reference method vs ACF: **a** Fitting of PCDD/Fs; **b** fitting of dl-PCBs. The box on the bottom right of each figure displays a zoom of the lower data. The axes' measuring units are arbitrary

The concentration (fg/m^3^) of each congener in the seven parallel samples was compared. Due to the large extent of concentrations (three orders of magnitude), data were normalized before the correlation. Figure 4 shows data concentrations of each congener in (fg/m^3^) from seven parallel samplings. In particular, Fig. 4 a reports the linear regression curve obtained by plotting the data of native PCDD/Fs congeners obtained using the ACF matrix vs. those obtained with the reference method (QFF + PUF), and Fig. 4 b reports the linear regression curve obtained by plotting the data sets obtained for PCB congeners. Each figure has a box on the bottom right that displays a zoom of the lower data.

As shown in these figures, a linear slope close to 1 was obtained for both in a wide range of concentrations. The correlation coefficients measured with native dl-PCBs (0.9943) and PCDD/Fs (0.9158) were high enough (Bland and Altman 2010) to let us state that the method using a single ACF matrix performed as well as the reference method using the QFF + PUF combination. The six “outlier” normalized data in each figure correspond to 2,3,7,8-TeCDF and PCB118, the common and dominant homologues in most combustion emissions.

### Advantages of the ACF method

Up to now, the method proposed with the ACF has been validated according to the ISO and EPA methods, and the absolute equivalence in the results concerning the double adsorbent system has been demonstrated. Comparing sampling on a single ACF matrix to the QFF + PUF combination also reveals a number of beneficial advantages. PUF suffers some oxidative degradation at high O3 levels in the environment, similar to many other organic polymeric adsorbents (Melymuk et al. 2017). Products resulting from PUF degradation can reduce the efficiency of the clean-up separation, leading to a lower signal-to-noise ratio in the GC–MS determination of PCDD/Fs and dl-PCBs. To a smaller extent, oxidation by O_3_ is also possible on some POPs deposited on the QFF thus increasing the uncertainty of PCDD/Fs and dl-PCBs determinations. These effects are largely prevented by the ACF as O_3_ is so rapidly reduced to O_2_ over a carbon surface as active carbon filters are commonly used in the O_3_ monitors to generate the zero levels in these instruments.

Another advantage offered by the ACF is the saving of solvent for the extraction of PCDD/Fs and dl-PCBs samples. It has been found that a Soxhlet apparatus with a smaller volume (100 mL) can be used with the ACF compared to the 250 mL one required with the QFF + PUF combination. While it is possible to perform 432 cycles in 36 h with a 250-mL Soxhlet apparatus, it is possible to perform 1080 cycles with a one having a volume of 100 mL. Since the extraction efficiency of a Soxhlet apparatus decreases exponentially as a function of the number of cycles, no substantial recovery of the sample occurs above a certain number of cycles. This means that it is possible to reduce the extraction time if the same number of cycles is used to extract PCDD/Fs and dl-PCBs from the ACF instead of the double system QFF + PUF. Reduction in solvent volumes and extraction times also produces a lower volume of wastes and a shorter exposure of the operator to chemicals making the use of the ACF safer. Since no backup adsorbents are required, high-volume sampling on the ACF is easier to handle, and material costs can be even lower than the QFF + PUF combination.

Actually, having more sampling devices, the volume of the extraction solvents increases as well as the materials that must be disposed of and of course the final cost of these analyses. The economic and time wasted tends to increase if each sampling device is extracted and analyzed in GC–MS separately. Even more important is that the use of several adsorption media introduces in the analysis a greater possibility of errors due to contamination and sample losses related to the operator’s ability during the various manipulations due to the sample processing steps that increase and to the matrix interferents coming from the materials themselves. Costs and time can be reduced as well as errors associated with the use of multiple capture media if a single sorbent is used to efficiently retain PCDD/Fs and dl-PCBs simultaneously in both the particulate and vapor phase.

## Conclusions

Briefly, the work was developed according to the following steps. First of all, the ability of ACF to retain both PCDD/Fs and dl-PCBs in the gas phase and the breakthrough limit for the different congeners was verified through dedicated experiments performed at increasing sampling volumes. Demonstrate the linearity of the method through R%s that meet the QA/QC from 3.5 up to 0.49 pg/m^3^ (1000 pg of SS solution from 288 to 2016 m^3^ sampled).

Based on the results obtained, the sampling system was adapted and tested for the simultaneous determination of PCDD/Fs and dl-PCBs in indoor and ambient air samples. Parallel analyses of polluted air samples collected using the ISO and US-EPA reference techniques proved the effectiveness, robustness, and accuracy of the ACF-based system.

The results obtained in this work show unequivocally that a single ACF matrix can be used for the simultaneous determination of PCDD/Fs and dl-PCBs in indoor and atmospheric samples satisfying all the QA/QC required by the ISO and EPA reference standard methods. The methods foresee a double sampling system consisting of a QFF for the particulate matter and a PUF for the gaseous phase; the proposed method is able to collect both phases while maintaining the same efficiency and with considerable advantages. Compared to the more widespread and used combined system, the ACF has no matrix effect and does not undergo atmospheric oxidation. It produces great advantages in terms of time and costs as well as being safer and versatile enough to be adapted to different commercially available samplers.

## Supplementary Information

Below is the link to the electronic supplementary material.

Supplementary file1 (DOCX 43 KB)

## Data Availability

The data used to support the findings of this study are available from the corresponding author upon request.
