# Supplementary material for: Particle and gas phase sampling of PCDD/Fs and dl-PCBs by activated carbon fiber and GC/MS analysis
Source: Environ Sci Pollut Res Int. 2023 Apr 20;30(24):65192–203. doi: 10.1007/s11356-023-27052-8 (PMC10182933; doi:10.1007/s11356-023-27052-8)
Supplement: Supplementary file 1 — Supplementary file1 (DOCX 43 KB) [file 11356_2023_27052_MOESM1_ESM.docx]

# PARTICLE AND GAS PHASE SAMPLING OF PCDD/Fs AND dl-PCBs BY ACTIVATED CARBON FIBER AND GC/MS ANALYSIS

Marina Cerasa^1^, Ettore Guerriero^1^, Catia Balducci^1^, Alessandro Bacaloni^2^, Piero Ciccioli^3^, Silvia Mosca*^1^

^1^Italian National Research Council, Institute of Atmospheric Pollution Research, Area della Ricerca di Roma 1, Montelibretti (RM), 00010, Italy

^2^Chemistry Department, Mathematics, Physics and Natural Sciences Faculty, Sapienza University Piazzale Aldo Moro, 5, Rome, 00185, Italy

^3^Italian National Research Council, Institute for Biological Systems, Area della Ricerca di Roma 1, Montelibretti (RM), 00010, Italy

(*) Corresponding Author: [silvia.mosca@iia.cnr.it](mailto:marina.cerasa@iia.cnr.it)

# Supplementary material

# Table S1 Schematic illustration of all the ISO 16000-13 and 14 and EPA-TO4A and TO9A methods steps

|  | **ISO 16000-13 and 14** | **EPA-TO 4A** | **EPA-TO 9A** |
| --- | --- | --- | --- |
|  | **PCDD/Fs and PCBs**  **indoor** | **PCBs**  **ambient air** | **PCDD/Fs**  **ambient air** |
| Sampling instrument | High-Vol Sampler | High-Vol Sampler | High-Vol Sampler |
| Sampled volume | No less than 50 m^3^ of air and should not exceed 360 m^3^ (24 h at 16 m^3^/h or 7 d at 2 m^3^/h).  The hourly air flow rate should not exceed 10 % of the air change rate of the room. If the air change rate is unknown, the air volume sampled per hour shall not exceed 10 % of the volume of the room. | Total volume equal to or greater than 300 m^3^ in 24 h | Total volume to sample 325 to 400 m^3^ in 24 h |
| Materials for sample Collection | Quartz Fiber Filter + PUF | Quartz Fiber Filter + PUF | Quartz Fiber Filter + PUF |
| Sample material preparation | Bake the quartz filters at 400°C for 5 h.  Wash PUF in Soxhlet apparatus with acetone for 24 h (~4 cycles/h) | Bake the quartz filters at 400°C for 5 h.  Wash PUF in a Soxhlet apparatus with acetone for 16 h (~4 cycles/h) | Bake the quartz filters at 400°C for 5 h.  Wash PUF in a Soxhlet apparatus acetone for 16 h (~4 cycles/h) |
| Extraction (Soxhlet) | The sampling media (filter + PUF) are combined and extracted together with Toluene or an equivalent solvent for 36h. | The sampling media (filter + PUF) are combined and extracted together with 10% diethyl ether in hexane for at least 16 h. | The sampling media (filter + PUF) are combined and extracted together with benzene for 16 h |
| Sampling standards | 100 μl of about 0,01 ng WHO-TEQ PCB/m^3^ (3600 pg of each compound). 100 µL of about 100 fg TEQ/m^3^ PCDD/Fs (25pg of Penta and Hexa; 50 pg of Hepta) for 180 m³ of sampling volume. | NO | 800 pg of ^37^Cl -2,3,7,8-TCDD or a mix with 4 ^13^C -labelled standards. |
| Extraction standards | 100 μl of about 0,01 ng WHO-TEQ PCB/m^3^ (3600 pg of each compound) 100 µL of about 100 fg TEQ/m^3^ PCDD/Fs (25 pg of Tetra, Penta and Hexa; 50 pg of Hepta and Octa) assuming 180 m³ of sampling volume | One or more surrogate compounds that have chemical structures and properties similar to those of the analytes of interest 100 ng of ^13^C -labeled Standard | 1000 pg of ^13^C -labeled Standard |

# Table S2 Standard solutions used for PCDD/Fs

| Congener | EN-1948SS  (pg/µL) | EN-1948ES  (pg/µL) | EN-1948IS  (pg/µL) |
| --- | --- | --- | --- |
|  |  |  |  |
| 1,2,3,7,8-Penta[^13^C_12_]DF | 200 | - | - |
| 1,2,3,7,8,9-Hexa[^13^C_12_]DF | 200 | - | - |
| 1,2,3,4,7,8,9-Hepta[^13^C_12_]DF | 400 | - | - |
|  |  |  |  |
| 2,3,7,8-Tetra[^13^C_12_]DD | - | 200 | - |
| 2,3,7,8-Tetra[^13^C_12_]DF | - | 200 | - |
| 1,2,3,7,8-Penta[^13^C_12_]DD | - | 200 | - |
| 2,3,4,7,8-Penta[^13^C_12_]DF | - | 200 | - |
| 1,2,3,4,7,8-Hexa[^13^C_12_]DD | - | 200 | - |
| 1,2,3,6,7,8-Hexa[^13^C_12_]DD | - | 200 | - |
| 1,2,3,4,7,8-Hexa[^13^C_12_]DF | - | 200 | - |
| 1,2,3,6,7,8-Hexa[^13^C_12_]DF | - | 200 | - |
| 2,3,4,6,7,8- Hexa[^13^C_12_]DF | - | 200 | - |
| 1,2,3,4,6,7,8-Hepta[^13^C_12_]DD | - | 400 | - |
| 1,2,3,4,6,7,8-Hepta[^13^C_12_]DF | - | 400 | - |
| Octa[^13^C_12_]DD | - | 400 | - |
| Octa[^13^C_12_]DF | - | 400 | - |
|  |  |  |  |
| 1,2,3,4-Tetrachloro[^13^C_12_]DD | - | - | 800 |
| 1,2,3,7,8,9-Hexachloro[^13^C_12_]DD | - | - | 800 |

# Table S3 Standard solutions used for dl-PCBs

| Congener |  | P-48SS  (pg/µL) | WP-LCS  (pg/µL) | WP-ISS  (pg/µL) |
| --- | --- | --- | --- | --- |
|  |  |  |  |  |
| 2,3,4,4'-Tetra[^13^C_12_]B | 60L | 100 | - | - |
| 3,3',4,5,5'-Penta[^13^C_12_]B | 127L | 100 | - | - |
| 2,3,3',4,5,5'-Hexa[^13^C_12_]B | 159L | 100 | - | - |
|  |  |  |  |  |
| 3,4,4',5-Tetra[^13^C_12_]B | 81L | - | 1000 | - |
| 3,3',4,4'-Tetra[^13^C_12_]B | 77L | - | 1000 | - |
| 2',3,4,4',5-Penta[^13^C_12_]B | 123L | - | 1000 | - |
| 2,3',4,4',5-Penta[^13^C_12_]B | 118L | - | 1000 | - |
| 2,3,4,4',5-Penta[^13^C_12_]B | 114L | - | 1000 | - |
| 2,3,3',4,4'-Penta[^13^C_12_]B | 105L | - | 1000 | - |
| 3,3',4,4',5-Penta[^13^C_12_]B | 126L | - | 1000 | - |
| 2,3',4,4',5,5'-Hexa[^13^C_12_]B | 167L | - | 1000 | - |
| 2,3,3',4,4',5-Hexa[^13^C_12_]B | 156L | - | 1000 | - |
| 2,3,3',4,4',5'-Hexa[^13^C_12_]B | 157L | - | 1000 | - |
| 3,3',4,4',5,5'-Hexa[^13^C_12_]B | 169L | - | 1000 | - |
| 2,3,3',4,4',5,5'-Hepta[^13^C_12_]B | 189L | - | 1000 | - |
| SYRINGE STANDARD |  |  |  |  |
| 2,3’,4’,5-Tetrachloro[^13^C_12_]B | 70L | - | - | 1000 |
| 2,3,3’,5,5’-Penthachloro[^13^C_12_]B | 111L | - | - | 1000 |
| 2,2’,3,4,4’,5’-Hexachloro[^13^C_12_]B | 138L | - | - | 1000 |
| 2,2’,3,3’,4,4’,5-Heptachloro[^13^C_12_]B | 170L | - | - | 1000 |

# Table S4 – Detailed information on data collection at “A. Liberti” monitoring station

|  | **date** | **avg T [°C]** | **min T [°C]** | **max T [°C]** | **Average Humidity [%]** | **Min Humidity [%]** | **Max Humidity [%]** | **Precip.** | **max wind [km/h]** |
| --- | --- | --- | --- | --- | --- | --- | --- | --- | --- |
| **24h** | 23/03/2016 | 14 | 11 | 16 | 56 | 36 | 76 | rain | 44 |
| **72 h** | 11/04/2016 | 17 | 7 | 21 | 54 | 27 | 93 | no | 13 |
|  | 12/04/2016 | 19 | 8 | 24 | 50 | 26 | 93 | no | 13 |
|  | 13/04/2016 | 21 | 15 | 26 | 47 | 21 | 73 | no | 28 |
| **136 h** | 26/04/2016 | 12 | 6 | 14 | 69 | 43 | 94 | rain | 20 |
|  | 27/04/2016 | 14 | 11 | 16 | 65 | 50 | 94 | rain | 20 |
|  | 28/04/2016 | 15 | 13 | 17 | 74 | 63 | 100 | rain | 20 |
|  | 29/04/2016 | 16 | 12 | 22 | 70 | 38 | 94 | no | 19 |
|  | 30/04/2016 | 16 | 10 | 21 | 72 | 49 | 94 | rain | 19 |
|  | 01/05/2016 | 13 | 9 | 18 | 80 | 55 | 100 | rain | 17 |
|  | 02/05/2016 | 12 | 7 | 8 | 78 | 52 | 100 | rain | 17 |

# Table S5 – Breakthrough tests 24h

The “*” means ^13^C-labelled

| **24 h** | **QFF** | **ACF A** | **ACF B** |
| --- | --- | --- | --- |
|  | **mean R%** | **mean R%** | **mean R%** |
| 2378-TeCDD* | 3.0 | 97 | 2 |
| 2378-TeCDF* | 2.7 | 95 | 3 |
| 12378-PeCDD* | 4.0 | 94 | 0.8 |
| 23478-PeCDF* | 5.0 | 95 | 2 |
| 123478-HxCDD* | 17 | 78 | 0.8 |
| 123678-HxCDD* | 18 | 72 | 0.8 |
| 123478-HxCDF* | 7.0 | 84 | 3 |
| 123678-HxCDF* | 10 | 89 | 0.8 |
| 234678-HxCDF* | 12 | 76 | 0.8 |
| 1234678-HpCDD* | 66 | 27 | 0.8 |
| 1234678-HpCDF* | 55 | 33 | 0.8 |
| OCDD* | 83 | 9.2 | 0.8 |
| OCDF* | 85 | 16 | 0.8 |
| 81L | 3.0 | 102 | 1.18 |
| 77L | 4.4 | 100 | 1.08 |
| 123L | 7.3 | 98 | 1.17 |
| 118L | 9.0 | 91 | 1.27 |
| 114L | 12 | 83 | <1 |
| 105L | 13 | 92 | <1 |
| 126L | 14 | 92 | <1 |
| 167L | 26 | 85 | <1 |
| 156L | 27 | 85 | <1 |
| 157L | 35 | 73 | <1 |
| 169L | 27 | 71 | <1 |
| 189L | 74 | 25 | <1 |

# Table S6 - Breakthrough tests 72h

The “*” means ^13^C-labelled

| **72 h** | **QFF** | **ACF A** | **ACF B** |
| --- | --- | --- | --- |
|  | **mean R%** | **mean R%** | **mean R%** |
| 2378-TeCDD* | 0.8 | 98 | 2.2 |
| 2378-TeCDF* | 1 | 114 | 4.0 |
| 12378-PeCDD* | 1.1 | 96 | 1.0 |
| 23478-PeCDF* | 4.3 | 92 | 1.0 |
| 123478-HxCDD* | 10 | 97 | 2.0 |
| 123678-HxCDD* | 13 | 83 | 1.0 |
| 123478-HxCDF* | 3 | 83 | 0.8 |
| 123678-HxCDF* | 6.2 | 77 | 0.8 |
| 234678-HxCDF* | 5.1 | 82 | 0.8 |
| 1234678-HpCDD* | 55 | 46 | 0.8 |
| 1234678-HpCDF* | 36 | 69 | 0.8 |
| OCDD* | 84 | 15 | 1.1 |
| OCDF* | 83 | 24 | 0.8 |
| 81L | 0 | 90 | 6.0 |
| 77L | 6.2 | 111 | 4.0 |
| 123L | 4.0 | 98 | 2.1 |
| 118L | 4.2 | 100 | 3.4 |
| 114L | 6.8 | 92 | 1.8 |
| 105L | 11 | 96 | 2.3 |
| 126L | 9.1 | 91 | 1.1 |
| 167L | 12 | 78 | 0.0 |
| 156L | 18 | 86 | 2.5 |
| 157L | 22 | 80 | 0.0 |
| 169L | 17 | 86 | 0.0 |
| 189L | 52 | 55 | 0.0 |

# Table S7 - Breakthrough tests 168h

The “*” means ^13^C-labelled

| **168 h** | **QFF** | **ACF A** | **ACF B** |
| --- | --- | --- | --- |
|  | **mean R%** | **mean R%** | **mean R%** |
| 2378-TeCDD* | 0.8 | 94 | 4 |
| 2378-TeCDF* | 0.8 | 103 | 7 |
| 12378-PeCDD* | 0.8 | 92 | 2 |
| 23478-PeCDF* | 0.8 | 91 | 4 |
| 123478-HxCDD* | 2 | 100 | 1 |
| 123678-HxCDD* | 1 | 101 | 0.8 |
| 123478-HxCDF* | 0.8 | 98 | 5 |
| 123678-HxCDF* | 3 | 89 | 3 |
| 234678-HxCDF* | 2 | 89 | 1 |
| 1234678-HpCDD* | 39 | 66 | 0.8 |
| 1234678-HpCDF* | 29 | 77 | 0.8 |
| OCDD* | 70 | 33 | 0.8 |
| OCDF* | 73 | 40 | 0.8 |
| 81L | 0 | 86 | 5.2 |
| 77L | 0 | 89 | 5.4 |
| 123L | 0 | 85 | 3.0 |
| 118L | 0 | 89 | 4.0 |
| 114L | 0 | 93 | 4.0 |
| 105L | 3.1 | 97 | 2.1 |
| 126L | 2.7 | 91 | 7.1 |
| 167L | 2.1 | 91 | 4.1 |
| 156L | 4.1 | 89 | 6.1 |
| 157L | 7.1 | 84 | 3.2 |
| 169L | 6.1 | 87 | 3.2 |
| 189L | 16 | 70 | 2.1 |
